# Effects of the COVID-19 pandemic on mental health - what do we know so far?

**DOI:** 10.1192/j.eurpsy.2021.839

**Published:** 2021-08-13

**Authors:** M. Marguilho, A. Nobre

**Affiliations:** Clínica 5, Centro Hospitalar Psiquiátrico de Lisboa, Lisboa, Portugal

**Keywords:** COVID19, mental, health, psychological

## Abstract

**Introduction:**

The devastating effects of the current pandemic are profoundly affecting peoples’s physical and psychological health. Numerous studies on the effects of previous infectious outbreaks have been published. Similarly, an increasingly growing body of research on COVID-19 has been developed and released, reporting a substancial psychological impact of both the outbreak and the response, suggesting that the population may express high levels of psychological symptoms.

**Objectives:**

This presentation aims to synthesize existent literature that reports on the effects of COVID-19 on psychological outcomes of the general population, groups with higher vulnerability and its associated risk factors.

**Methods:**

Bibliographic research was made through scientific databases such as PubMed and EMBASE. No time limit was used. Pertinent articles were carefully reviewed for additional relevant citations.

**Results:**

Generally, there is a higher prevalence of symptoms of adverse psychiatric outcomes among the public when compared to the prevalence before the pandemic. Psychological reactions to pandemics include maladaptive behaviours, emotional distress and symptoms of stress, anxiety, depression, and avoidance behaviors. The groups known to be at higher risk for mental health problems during the pandemic are: women, healthcare workers, people under 40 years old and with chronic diseases. Other risk factors are: frequent exposure to social media/news relating to COVID-19, poor economic status, lower education level, and unemployment.

**Conclusions:**

The COVID-19 pandemic represents an unprecedented threat to mental health. In addition to flattening the curve of viral transmission, special attention needs to be paid to the challenges it poses to the mental health of the population at a global scale.

